# Three *Aedes* species infested by mermithids in France[Fn FN1]

**DOI:** 10.1051/parasite/2023013

**Published:** 2023-04-07

**Authors:** Jean-Philippe Martinet, Issam Aatif, Jérôme Depaquit

**Affiliations:** 1 Université de Reims Champagne-Ardenne, Faculté de Pharmacie, ANSES, SFR Cap Santé, EA7510 ESCAPE – USC VECPAR 51 rue Cognacq-Jay 51096 Reims CEDEX France; 2 Institut Pasteur, Department of Virology, Arboviruses and Insect Vectors 25–28 Rue du Dr Roux 75015 Paris France

**Keywords:** Mermithids, Mosquito, *Aedes*, France

## Abstract

Mermithid nematodes (Nematoda, Mermithidae) parasitising mosquitoes could be useful as biological agents for the control of host populations. Nine female mosquitoes belonging to the species *Aedes cantans*, *Ae. communis,* and *Ae. rusticus* were found parasitised by mermithids in Northern France. Sequencing of partial 18S rDNA showed 100% sequence homology for all processed specimens. The mermithid sequences were closely related to specimens previously recorded from *Anopheles gambiae* in Senegal. However, 18S sequences do not allow for identification of nematodes at the genus or species level. Our specimens could also be related to *Strelkovimermis spiculatus*, or belong to other genera not yet sequenced such as *Empidomermis,* the only mermithid genus recorded from mosquitoes in France.

## Abstract (fr)

Les nématodes mermithidés (Nematoda, Mermithidae) parasitant les moustiques pourraient être utiles comme agents biologiques pour contrôler les populations hôtes. Neuf femelles de moustiques appartenant aux espèces *Aedes cantans*, *Ae. communis* et *Ae. rusticus* ont été trouvées parasitées par des mermithidés dans le nord de la France. Le séquençage de l’ADNr 18S partiel a montré une homologie de séquence de 100 % pour tous les échantillons traités. Les séquences des mermithidés étaient proches de spécimens précédemment signalés chez *Anopheles gambiae* au Sénégal. Les séquences 18S, cependant, ne permettent pas l’identification des nématodes au niveau du genre ou de l’espèce. Nos spécimens pourraient également être apparentés à *Strelkovimermis spiculatus*, ou appartenir à d’autres genres non encore séquencés comme *Empidomermis*, le seul genre de mermithidés recensé chez les moustiques en France.

## Introduction

Mermithids nematodes are obligate endoparasites of Arthropoda, especially insects. They are considered host specific, usually at the host-species or at the family-species level [17]. Species parasitising mosquitoes have probably been the most studied, as they can be used as biological agents to control their host populations [1, 6, 15, 16]. The presence of mosquito mermithids in France is poorly known, except for the description of a new species isolated from *Aedes detritus* in Southern France [7]. During a research program related to mosquito arboviruses, mosquitoes were sampled in Northern France. We report here the presence of mermithids in three different mosquito species analyzed by partial 18S ribosomal DNA sequencing.

## Materials and methods

### Mosquito sampling

During a three-year epidemiological program focusing on mosquito arboviruses, mosquito larvae were episodically collected to create a qualitative inventory and assess the feasibility of establishing laboratory colonies of local mosquitoes for vector competence experiments. To this end, mosquito larvae were sampled in 2019 in two selected localities in north-eastern France: Berru on April 1st (49.267533 N, 4.133583 E) and in the vicinity of the Der-Chantecoq Lake on April 16th (48.576553 N, 4.692353 E).

Water puddles located in sylvatic environments were sampled by hand using a deeper. Collected larvae were placed in jars containing water from the local environment, then immediately transported to the insectary and placed in two labelled cages before emergence of adults. Larvae were fed yeast pellets and maintained at 22 °C. Adults were maintained at 22 °C, 60% relative humidity and given free access to a 10% sucrose solution. Each population was monitored daily in the laboratory. Mermithid infestation was characterized by the emergence of parasites escaping from adult mosquitoes.

### Mosquito processing

Emerged mosquitoes were anesthetized by cold, and morphologically identified at the species level using the MosKeyTool taxonomic key [9].

Legs were used for molecular identification. DNA was extracted with a DNeasy Blood and Tissue extraction kit (Qiagen, Hilden, Germany), following the manufacturer’s instructions. Polymerase chain reaction was performed on a 648 bp fragment of the COI gene using primers LEPF1 (5′–TTTCTACAAATCATAAAGATATTGG–3′) and LEPR1 (5′–TAAACTTCTGGATGTCCAAAAAATCA–3′), according to experimental conditions found in the literature [10, 13, 18].

### Mermithid processing

The anterior and posterior parts of each worm were cut off and cleared in Amman lactophenol between the slide and cover slide. These specimens are available upon request to the authors. Pictures were taken using Stream Essentials^©^ software version 1.7 and a DP-26 video camera connected to a SZX10 stereomicroscope (Olympus, Tokyo, Japan).

Genomic DNA was extracted from the middle part of the worm. Molecular identification of mermithids was performed by amplification and sequencing of partial 18S rDNA thanks to cycles and primers Merm forward 5′–CAAGGACGAAAGTTAGAGGTTC–3′ and Merm reverse 5′–GGAAACCTTGTTACGACTTTTA–3′ as proposed by Kobylinski *et al.* [12].

To amplify COI mtDNA, several couples of primers were used as described in the literature [19], including JB3 (5′–TTTTTTGGGCATCCTGAGGTTTAT–3′) and JB4.5 (5′–TAAAGAAAGAACATAATGAAAATG–3′) [11]. Due to the lack of amplification, we designed three pairs of consensus primers by alignment of sequences from other mermithids obtained from GenBank. The in-house designed forward primers were 5′–ARAACAAAATGAAAGTG–3′; 5′–AGTTAATAACATAGTAATAGC–3′; 5′–ACKACAAARTAKGTRTCATG–3′. The in-house designed reverse primers were 5′–ATTYTWCCTGYBTTTGG–3′; 5′–CCTGARGTWTAYRTWYTAATT–3′; 5′–ATAATTTTTTTTATRGTTATACC–3′. All these primers were combined and tested at hybridization temperatures ranging from 40 °C to 55 °C.

### Molecular analysis

Amplicons were sequenced through Sanger technology (Genewiz, Leipzig, Germany). First, mosquito and mermithid sequences (Table 1) were compared to existing GenBank sequences with the BLAST algorithm [2] and mosquito identification was considered accurate when similarity was higher than 99%. Second, mermithid sequences were edited and aligned using Muscle software [8]. The GTR+G model of molecular evolution was determined with ModelTest-NG [5] and the phylogenetic tree was constructed using the maximum likelihood (ML) method in MEGA11 [20].

**Table 1:** Mosquitoes, their mermithids and sequences.

Specimen voucher	Collection locality	Specimen gender	Identification	Mosquito COI GenBank accession numbers	GenBank homology	Closest GenBank sequence	Number of mermithids parasitising the mosquito	GenBank for new 18S sequences of mermithids
BR2	Berru	Female	*Aedes cantans*	OQ244837	100%	MK403102	2	OQ249540
BR4	Berru	Female	*Aedes communis*	OQ244838	100%	MT149922	1	OQ249541
BR5	Berru	Female	*Aedes cantans*	OQ244839	100%	MK403531	2	OQ249538, OQ249539
BR9	Berru	Female	*Aedes cantans*	OQ244840	100%	MK403516	1	OQ249542
BR11	Berru	Female	*Aedes cantans*	OQ244841	100%	MK403516	1	OQ249543
DER 5,6 G2	Der’s lake	Female	*Aedes rusticus*	OQ244843	99.67%	MK403533	2	OQ249533
DER 5,6 G3	Der’s lake	Female	*Aedes rusticus*	OQ244844	100%	MK403533	2	OQ249544
DER 5,6 G4	Der’s lake	Female	*Aedes rusticus*	OQ244845	100%	MK403533	4	OQ249534, OQ249535
DER 5,6 Arg	Der’s lake	Female	*Aedes rusticus*	OQ244842	100%	MK403533	2	OQ249536, OQ249537

## Results

### Mosquitoes

Mosquito larvae collected in Berru included 45 females (12 *Aedes cantans*, 1 *Ae. communis* and 32 *Ae. rusticus*). Samples collected in the Der-Chantecoq lake included 55 *Ae. rusticus* females. As the first evidence of parasitism was observed on female mosquitoes at a time when males were already discarded from the cages, we do not possess data concerning Mermithid infestation in male mosquitoes.

Five female mosquitoes from Berru were infested by mermithids: four *Ae. cantans* and one *Ae. communis*.

**Figure 1:**
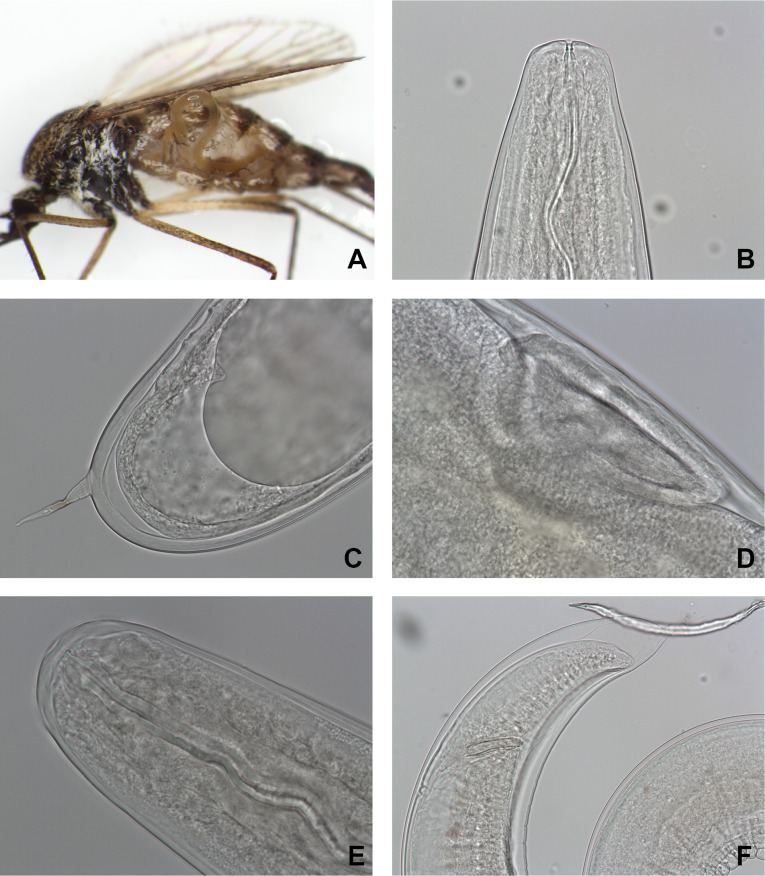
*Aedes rusticus* parasitized by a mermithid (A) and microphotographs of mermithid specimens isolated from *Ae. rusticus*. B, E: head; C, F: tail showing details, exhibiting a spur; D: vagina.

Four female mosquitoes from the Der-Chantecoq lake were infested by mermithids (Fig. 1).

The identification of the infested mosquitoes was based upon morphological characters and by sequencing showing more than 99.6% of similarity with the reference sequences (MK403102, MK403531 and MK403516 for *Ae. cantans*; MT149922 for *Ae. communis*; MK403533 for *Ae. rusticus*).

Sequences of mosquitoes collected in the present study are available in GenBank under accession numbers OQ244837–OQ244845.

### Mermithids

Observation of the parasite juvenile stages revealed a posterior part with a straight spur (Fig. 1).

Sequences of 776 bp were obtained on the processed specimens. We analyzed an alignment of 750 bp of partial 18S rDNA sequences in order to compare our sequences with homologous ones available in GenBank. The sequences obtained from the mermithids isolated from the nine infected females were all identical (100% homology: no variability observed in the specimens processed in the present study). They are available in GenBank under accession numbers OQ249533–OQ249544.

**Figure 2:**
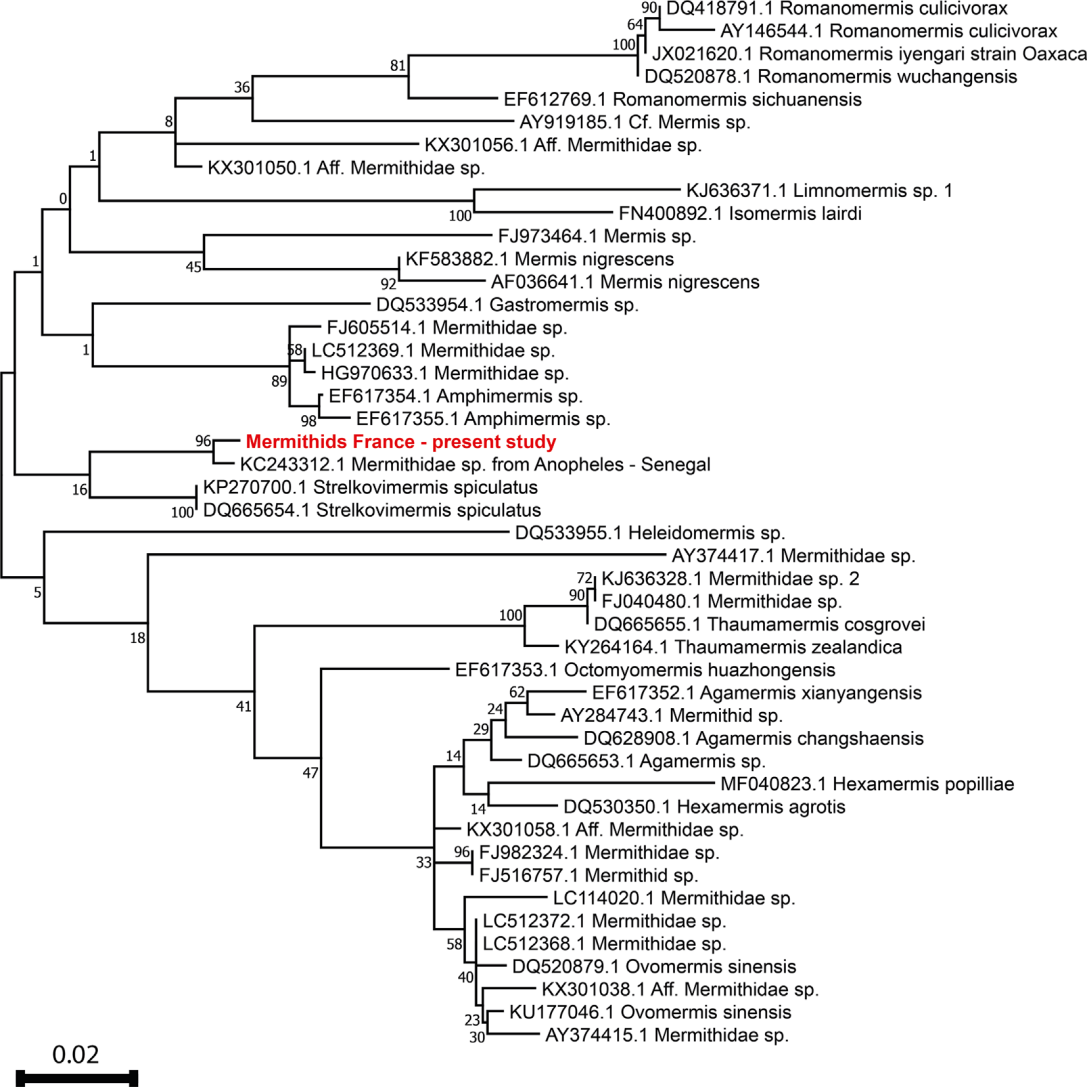
Maximum-likelihood tree based on partial 18S rDNA sequences available in GenBank, including the sequence of mermithids processed in the present study (in red). Bootstrap values are indicated on specific branches.

The BLAST analysis showed that the closest sequence is that of a Mermithidae sp. isolated in some *Anopheles gambiae* from Senegal (99.21% homology with sequence KC243312 obtained by comparison of 756 bp of JOSN1 showing 6 variable and 750 conserved positions out of a total of 756 compared nucleotides) followed by several sequences of *Strelkovimermis spiculatus* (95.67% homology, meaning 729 conserved and 33 variable positions out of a total of 762). The ML tree obtained is shown in Figure 2.

We were, however, unable to amplify the COI domain of the mermithids with both published and in-house designed primers.

## Discussion

Reliable morphological identification of mermithids must be performed on adults which constitute a free stage of these parasites. Unfortunately, we were not able to observe such stages and thus could not reliably identify the parasites collected. According to Nickle, they could belong to several genera (*Reesimermis*, *Perutilimermis*) [14].

According to 18S rDNA sequences, the specimens most closely related to ours were those isolated from Senegalese malaria vector mosquitoes *Anopheles gambiae* [12], which remained unidentified according to i) the difficulty in identifying parasites stages using morphological characters, and ii) the lack of a match with other sequences available in GenBank.

The specimens we collected and processed exhibited 6 mutations (99.21% homology) when compared to these Senegalese mermithids (GenBank accession number KJ636371). Considering that 18S rDNA is a highly conserved molecular marker, we cannot conclude regarding the exact identification of our samples at a species nor genus level. Studies carried out on triatomine bugs [4] as well as one study carried out on the digenean *Fasciola hepatica* [3], estimated that the conventional molecular clock rate is 1.8 × 10^−10^ substitutions per site per year (1.8% per 100 my) for the evolution of the 18S gene. If this calibration is accurate, the divergence time between Senegalese and French mosquito specimens would be close to 100 million years. Unfortunately, we were not able to amplify mermithid COI mtDNA, despite several repeats and the use of published and in-house designed primers. Nonetheless, based on the 100% homology of sequences between our samples, we can only conclude that they should belong either to the same species or to a very closely related one. The absence of *Empidomermis*, *Culicimermis*, *Hydromermis*, or *Perutilimermis* 18 S rDNA sequence entries in GenBank does not allow reliable identification down to the species level.

To date, *Strelkovimermis* samples (closest species identified in GenBank with 95.67% homology with our samples) have never been collected in France. To our knowledge, the only available report of a mermithid nematode in French mosquitoes is related to the original description of *Empidomermis riouxi* Doucet, Laumond & Bain, 1979 from *Aedes detritus* in Southern France [7]. We cannot exclude, based on our results, that the specimens processed in the present study could belong to this species.

The paucity of available data prohibits positive identification of the parasites we processed as previously encountered in the mermithid parasitism of bees or black flies [19, 21]. Considering the importance of these nematodes in the biocontrol of mosquitoes, our work will, however, provide some information for future investigations on mermithids. Repeated and large-scale use of current vector control strategies based on long lasting insecticide net distribution and indoor residual spraying of insecticides has led to an increased prevalence of mosquito resistance. Similarly, excessive insecticide use in agriculture has led to environmental pollution with an ecological impact on fauna and flora. New and innovative control strategies, such as the use of mermithids as biological agents to fight vector-borne diseases, remain to be explored.
